# Individual vs. Group Delivery of Acupuncture Therapy for Chronic Musculoskeletal Pain in Urban Primary Care—a Randomized Trial

**DOI:** 10.1007/s11606-019-05583-6

**Published:** 2020-02-19

**Authors:** M. Diane McKee, Arya Nielsen, Belinda Anderson, Elizabeth Chuang, Mariel Connolly, Qi Gao, Eric N Gil, Claudia Lechuga, Mimi Kim, Huma Naqvi, Benjamin Kligler

**Affiliations:** 1 Department of Family and Social Medicine, Albert Einstein College of Medicine, New York, USA; 2 Department of Family Medicine and Community Health, University of Massachusetts Medical School, Worcester, USA; 3 Department of Family Medicine & Community Health, Icahn School of Medicine at Mount Sinai, New York, USA; 4 Pacific College of Oriental Medicine, Chicago, USA; 5 Institute of Clinical and Translational Research, Albert Einstein College of Medicine, New York, USA; 6 Department of Rehab Medicine, Albert Einstein College of Medicine, New York, USA; 7 Integrative Health Coordinating Center , U.S. Veterans Health Administration, Washington, D.C., USA

**Keywords:** pain, acupuncture therapy, health disparities, integrative medicine

## Abstract

**Background:**

Acupuncture has been shown to be effective for the treatment of chronic musculoskeletal back, neck, and osteoarthritis pain. However, access to acupuncture treatment has been limited in medically underserved and low-income populations.

**Objective:**

Acupuncture therapy delivered in groups could reduce cost and expand access. We compared the effectiveness of group versus individual acupuncture for pain and function among ethnically diverse, low-income primary care patients with chronic musculoskeletal pain.

**Design:**

This was a randomized comparative effectiveness non-inferiority trial in 6 Bronx primary care community health centers. Participants with chronic (> 3 months) back, neck, or osteoarthritis pain were randomly assigned to individual or group acupuncture therapy for 12 weeks.

**Participants:**

Seven hundred seventy-nine participants were randomized. Mean age was 54.8 years. 35.3% of participants identified as black and 56.9% identified as Latino. Seventy-six percent were Medicaid insured, 60% reported poor/fair health, and 37% were unable to work due to disability.

**Interventions:**

Participants received weekly acupuncture treatment in either group or individual setting for 12 weeks.

**Main Measures:**

Primary outcome was pain interference on the Brief Pain Inventory at 12 weeks; secondary outcomes were pain severity (BPI), physical and mental well-being (PROMIS-10), and opiate use. Outcome measures were collected at baseline, 12 and 24 weeks.

**Key Results:**

37.5% of individual arm and 30.3% in group had > 30% improvement in pain interference (*d* = 7.2%, 95% CI − 0.6%, 15.1%). Non-inferiority of group acupuncture was not demonstrated for the primary outcome assuming a margin of 10%. In the responder analysis of physical well-being, 63.1% of individual participants and 59.5% of group had clinically important improvement at 12 weeks (*d* = 3.6%, 95% CI − 4.2%, 11.4%).

**Conclusions:**

Both individual and group acupuncture therapy delivered in primary care settings reduced chronic pain and improved physical function at 12 weeks; non-inferiority of group was not shown.

**Trial Registration:**

Clinicaltrials.gov # NCT02456727

**Electronic supplementary material:**

The online version of this article (10.1007/s11606-019-05583-6) contains supplementary material, which is available to authorized users.

## INTRODUCTION

The prevalence of chronic pain conditions in the adult US population ranges from 11 to 47% in large surveys^1–8^; low back and neck pain, osteoarthritis (OA), and headache are the most common.^9^ Underserved and ethnically diverse populations are especially at risk for pain and pain undertreatment,^5, 10–12^ and these disparities are compounded when limited English proficiency impacts communication.^5^ Living with chronic pain is associated with impairment of physical and psychological functioning,^13–15^ lost productivity,^16^ and lower socioeconomic status.^6^

Acupuncture therapy is effective in the treatment of chronic pain conditions^17–20^ including chronic low back pain,^21–24^ neck pain,^24–26^ and knee pain from osteoarthritis.^27–32^ A recently updated individual patient data meta-analyses including over 20,000 patients with chronic pain showed acupuncture to be significantly better than sham treatment or usual care with only a 15% reduction in treatment effect at 1 year.^33^ Acupuncture therapy is supported or recommended as part of comprehensive pain care^12^ by the Agency for Healthcare Research and Quality (AHRQ),^34^ the American College of Physicians (ACP),^35^ and the Joint Commission (TJC).^36, 37^

Acupuncture therapy has been predominantly studied in the individual setting^17^; it has been shown to be effective and feasible for low-income, ethnically diverse, chronic pain patients delivered in community health center settings.^38–40^ However, lack of insurance coverage and limited access pose barriers to implementation in this population. To reduce cost, increase access, and meet patient demand, group acupuncture therapy is now being offered across the USA. In group acupuncture, patients are treated simultaneously, in a staggered fashion, situated near and in view of one another. Studies demonstrate that group acupuncture is acceptable to patients,^38, 41–46^ and early studies show it to be effective for pain.^46^ However, to date, no studies have compared the effectiveness of group versus individual acupuncture for chronic pain.^47,^^48^ The “Acupuncture Approaches to Decrease Disparities in Outcomes of Pain Treatment Two Arm Comparative Effectiveness Trial” (AADDOPT-2) sought to answer this question in an underserved and ethnically diverse patient population.

## METHODS

### Design Overview

AADDOPT-2 was a randomized, non-blinded comparative effectiveness trial. All participants were referred for acupuncture therapy by primary care providers (PCPs). The study consisted of 12 weekly sessions (treatment phase) and a 12-week follow-up phase. The primary hypothesis was that group was non-inferior to individual treatment for improving pain interference. Participants were recruited between May 2015 and August 2017. The Institutional Review Board of Albert Einstein College of Medicine approved the study.

### Study Setting

Participating primary care practices are located in the Bronx, NY, where 85.5% of residents are from an ethnic minority of whom more than half (56.7%) are Hispanic. Nearly a third of the population lives below poverty level. The six practices provide comprehensive primary care; 5 of the 6 are federally qualified health centers.

### Participants

We enrolled adults aged > 21 who received primary care at a participating health center and had (1) a diagnosis of chronic pain (> 3 months) due to osteoarthritis of any joint, or chronic neck or back pain related to non-cancer diagnoses; (2) fluency in English or Spanish; (3) ability to provide a phone number; and (4) intent to be available for up to 24 weeks. Exclusions were current anticoagulant use, and inability to provide informed consent due to mental illness or cognitive impairment. No minimum pain score was required for inclusion.

### Interventions

Participants in both arms continued to receive usual care for management of chronic pain. Usual care included medical diagnostic evaluation, analgesic drug therapies, recommendations for physical activity, and sometimes referral to specialist physicians or physical therapy. A detailed description of the development and implementation of our acupuncture manualization has been published separately.^49^ We deployed a team of 6 licensed acupuncturists who treated patients on-site in 5 of the 6 participating health centers; for one site, participants were treated at a health center nearby due to space constraints. All of the acupuncturists delivered both group and individual acupuncture sessions. The protocol employed “responsive manualization,” a pragmatic approach that allows for individualizing treatment from a consensus-built array of options.^50^ The manual had a common set of acupuncture points with optional points and techniques allowing treatments to be responsive to the heterogenous and evolving nature of an individual’s condition. All treatment followed guidelines for safety and correct methodology.^51^ Reporting followed Standards for Reporting Interventions in Clinical Trials of Acupuncture (STRICTA).^52^ In addition to acupuncture needling, the manual also provided for the incorporation of therapies often used with acupuncture including palpation,^53, 54^ Tui na (a traditional Chinese manual therapy),^55, 56^ Gua sha (unidirectional press stroking of a lubricated area of skin with a smooth, round-edged instrument),^46, 57, 58^ and extended auricular treatment with ear seeds (*Semen Vaccaria*).^59–62^ Participants were also given general lifestyle recommendations in terms of diet, the importance of moving, and external hot and cold exposure.

### Randomization and Treatment Arms

The randomization scheme was computer generated by the study statistician using a random number generator in the SAS software system. Randomization was stratified by source of pain: back pain versus other pain with block sizes of 2–4. The allocation was held by a supervisory staff member who had no contact with participants. Study allocation was not visible to enrolling staff, or provided to the patient, until baseline data was collected. Due to variable wait lists, the range of time from randomization to starting acupuncture was 0–311 days (mean 26 days). Individual acupuncture sessions were scheduled on the half hour with the acupuncturist simultaneously working 2 exam rooms. Group participants received treatment in a setting with up to 6 patients in the group at any one time, seated in chairs in a large room (conference or multi-use rooms). Initial treatments in group were scheduled every 20 min, and follow-up treatments every 15 min. The acupuncturist was present throughout the entire treatment period and could also adjust or add treatment. Patients could lean and rest forward on a table to allow access to the dorsal body.

### Outcomes and Follow-up

All measures were administered via phone in English or Spanish. Participants did not receive an incentive to attend acupuncture treatments but did receive modest incentives to complete the research interviews. The baseline research interview was conducted immediately prior to randomization, including demographics and a measure of depressive symptoms, the Patient Health Questionnaire (PHQ-9).^63^ The primary outcome was defined as > 30% improvement in pain interference (defined as “the self-reported consequences of pain on relevant aspects of a person’s life [including] the extent to which pain hinders engagement with social, cognitive, emotional, physical, and recreational activities”)^64^ between baseline and week 12 as measured by the *Brief Pain Inventory: Short Form* (*BPI*)*.*^65,^^66^, A recent review confirms that 30% improvement in pain represents clinically important change.^67^ Secondary outcomes included pain severity on the BPI and quality of life measured by the 10-item Patient-Reported Outcomes Measurement Information Systems (*PROMIS-10*) global health measure, which includes ratings of physical function and emotional distress.^64, 68^ We tracked use of *opiate medications* using two methods. Patients were asked at baseline, 12 and 24 weeks if they had a prescription for an opiate pain reliever from a physician, and if so, the number of days used in the last week. In addition, we extracted prescriptions for opiates written and refilled directly from the electronic medical record (EMR, EPIC ^tm^) using EMR extraction software (Clinical Looking Glass™; Emerging Health Information Technology; Yonkers, NY). Patient-reported outcomes were assessed at baseline, 12 and 24 weeks; the 24-week time point allowed assessment of maintenance of intervention effects.

### Statistical Methods

The study was designed to evaluate whether group was non-inferior to individual acupuncture for improving pain. The primary outcome was response to treatment, as defined by a 30% or greater improvement on the BPI pain interference measure between baseline and 12 weeks. The margin of non-inferiority was defined as an absolute difference of *δ* = 10% (individual–group) in the proportion of patients who responded to treatment. With a sample size of 282 subjects per group, the study had 80% power with a one-sided *α* = 2.5% to conclude that group therapy is non-inferior to individual therapy assuming the true response rate in both groups is 35%. To account for a 20% loss to follow-up rate, the target enrollment was 350 patients per arm.

Analyses were conducted according to the intent-to-treat (ITT) approach, followed by the per protocol (PP) method. The difference in pain interference response rates between the treatment arms was estimated along with corresponding two-sided 95% confidence intervals. Non-inferiority of the group approach relative to the individual approach was declared if the upper limit of the 95% confidence interval for the true difference in response rates (individual therapy rate–group therapy rate) was less than *δ*, the margin of non-inferiority. Stratified analysis using the Cochran-Mantel-Haenszel method was also performed to adjust for the randomization stratification factor, source of pain. Since unstratified and stratified results were nearly identical, only the former are reported. In addition, pain interference as measured on the original continuous scale was also analyzed by fitting analysis of covariance models with treatment group and baseline pain interference value as predictor variables.

Secondary outcomes, pain severity and global health, were analyzed using similar approaches. For opiate analgesic use, we conducted one EMR data extract for all opiate prescriptions for each participant, 6 months after the final participant-initiated treatment. Standard conversions were used to calculate oral morphine equivalents from extracted prescriptions.^69, 70^ Quantities were assumed to cover a 30-day supply unless otherwise specified by the prescriber. Participants were assumed to be taking all available as needed or PRN doses every day. For subjects with at least one opioid analgesic prescription during the study period, the average daily dose of opioids was compared over the 12 weeks pre-randomization and weeks 4–16 post-randomization using the Wilcoxon signed-rank test (the 4 weeks immediately following randomization were omitted to allow time for the patient to begin treatment and receive dosing adjustments from their prescriber). The same analysis was repeated for 24 weeks pre-randomization compared with 4–28 weeks post-randomization. Proportions of patients with an opioid prescription were compared across the same periods using McNemar’s test.

#### Handling of Missing Data

Both list-wise deletion and multiple imputation (MI) using chained equations were applied to address missing data and yielded similar findings. Details and results of the MI approach are in the supplementary table c.

A complete description of the study protocol and statistical analysis approach will be available in our final study report on the PCORI website in May 2020.^71^

## RESULTS

### Participant Flow

**Figure 1:**
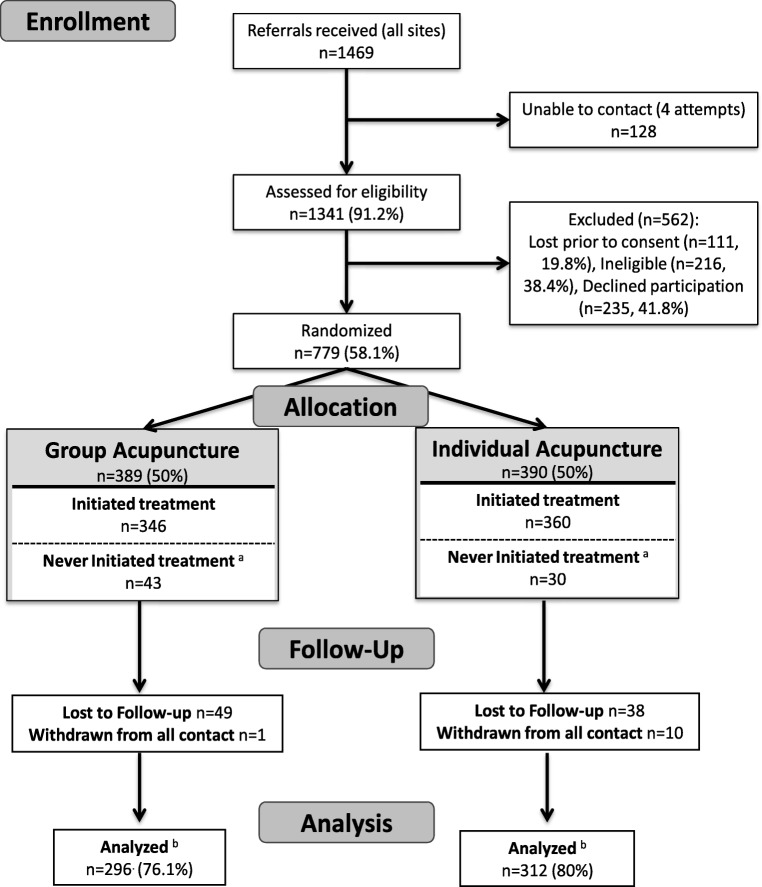
Participant flow in AADDOPT-2 (CONSORT diagram). a No further data collected; b Available for analysis at 12 weeks for primary outcomes (ITT).

Of 1469 referrals received, 1341 (91.3%) were screened. Of screened individuals, 41.9% either declined participation, were lost to follow-up prior to randomization, or were ineligible. We randomized 779 to group (*n* = 389) or individual (*n* = 390) arms; 73 (9.4%) of individuals who were eligible completed baseline data and were randomized but never initiated acupuncture. In many cases, these subjects were initially on a wait list for treatment but subsequently declined to participate when an opening became available. We scheduled 2.8 patients per hour in group and 2.0 in individual sessions. Our actual average number of patients seen per hour was 1.9 for group and 1.4 for individual; this accounts for set up and break down time as well as no-shows (Fig. 1).

Rates of loss to follow-up (i.e., those with no follow-up data collected) were similar in the two arms at 12 weeks (individual 12.3% and group 12.8%). There were no discernible patterns of skipped responses. The proportion of participants with either no survey or skipped questions resulting in insufficient data to calculate the pain outcomes (pain interference and pain severity) at each assessment point is provided in Supplementary Table a. Demographic characteristics of those with and without missing primary outcome (pain interference) at 12 weeks are also shown in Supplementary Table b. Missing pain interference data was more common among those with high school education or less, and the mean age of participants with missing data was 52.8 years compared with 55.6 for those without missing data.

### Participant Baseline Characteristics

For the overall sample (*n* = 779; see Table 1), the mean age was 54.8 years. Participants identified as black (35.3%), white (13.4%), and multiracial (12.3%). Over half identified as Latino (56.9%); 76% were Medicaid insured, 60% reported poor/fair health, and 37% were unable to work due to disability. Participants had a baseline pain interference score of 6.1. One-quarter (26.0%) reported having a prescription for an opiate pain reliever. Group and individual arm participants did not differ with regard to demographics and baseline measures. None of the key potential confounders were significantly different across treatment arms.

**Table 1:** Participant Baseline Demographics

Demographic variable	Group (*n* = 389)	Individual (*n* = 390)	*p* value	Total (*n* = 779)
Age, mean (SD)	54.2 (13.8)	55.4 (13.2)	0.19	54.8 (13.5)
Sex (*n* (%))			0.73	
Female	315 (81.0%)	312 (80.0%)		627 (80.5%)
Male	74 (19.0%)	78 (20.0%)		152 (19.5%)
Spoken language (*n* (%))			0.9*	
English	292 (78.7%)	290 (78.0%)		582 (78.3%)
Spanish	79 (21.3%)	81 (21.8%)		160 (21.5%)
Other	1 (0.3%)	0 (0.0%)		1 (0.1%)
Ethnicity (*n* (%))			0.34*	
Hispanic/Latino(a)	219 (56.3%)	224 (57.6%)		443 (56.9%)
Non-Hispanic	168 (43.2%)	159 (40.9%)		327 (42%)
Do not know	2 (0.5%)	6 (1.5%)		8 (1.0%)
Race (*n* (%))			0.24*	
American Indian/Native	15 (4.0%)	22 (5.8%)		37 (4.9%)
Asian	6 (1.5%)	4 (1%)		10 (1.3%)
Black/African American	141 (36.3%)	134 (34.4%)		275 (35.3%)
White	46 (11.8%)	58 (14.9%)		104 (13.4%)
Multiracial	43 (11.1%)	53 (13.5%)		95 (12.3%)
Other	138 (35.5%)	119 (30.5%)		257 (33%)
Working status (*n* (%))			0.24	
Unable to work due to disability	143 (36.8%)	147 (37.7%)		290 (37.2%)
Unemployed	58 (14.9%)	44 (11.3%)		102 (13.1%)
Other (employed, retired, etc.)	188 (48.3%)	199 (51%)		387 (49.7%)
Household income support (*n* (%))			0.40	
Any Support	114 (29.7%)	103 (27.0%)		217 (28.3%)
No Support	270 (70.3%)	279 (73.0%)		549 (71.7%)
Receive SSI (*n* (%))			0.47	
Yes	177 (45.5%)	217 (55.6%)		394 (50.6%)
Health insurance (*n* (%))			0.78	
Medicaid	301 (77.4%)	288 (73.8%)		589 (75.6%)
Private	76 (19.5%)	88 (22.6%)		164 (21.1%)
None	8 (2.1%)	8 (2.1%)		16 (2.1%)
Other	4 (1.0%)	6 (1.5%)		10 (1.3%)
Annual income (*n* (%))			0.37	
< $20,000	183 (47.0%)	179 (45.9%)		362 (46.5%)
$20,000–$39,999	81 (20.8%)	74 (19%)		155 (19.9%)
> $40,000	42 (10.8%)	57 (14.6%)		99 (12.7%)
Do not know/refused	83 (21.3%)	80 (20.5%)		163 (21%)
Opioid prescription by self-report (*n* (%))				
Yes	106 (27.8%)	93 (24.2%)	0.26	199 (26.0%)
Opioid prescription in EMR (*n* (%))				
Yes				
Mean opioid MME				
Referring condition (*n* (%))				
Back pain	261 (67.1%)	273 (70.0%)	0.38	534 (68.5%)
Neck pain	70 (18.0%)	68 (17.4%)	0.84	138 (17.7%)
Osteoarthritis	94 (24.2%)	112 (28.7%)	0.15	206 (26.4%)
2 or more	77 (22.5%)	91 (25.7%)	0.33	168 (24.1%)
PHQ-9 score, mean (SD)	8.75 (6.02)	8.74 (6.07)	0.98	8.74 (6.04)
Overall PROMIS Global Health Score (*n* (%))			0.95	
Poor	80 (20.6%)	85 (21.8%)		165 (21.2%)
Fair	152 (39.1%)	145 (37.3%)		297 (38.2%)
Good	111 (28.5%)	104 (26.7%)		215 (27.6%)
Very good	33 (8.5%)	42 (10.8%)		75 (9.6%)
Excellent	13 (3.3%)	13 (3.3%)		26 (3.3%)

*Fisher’s exact test

### Participation in the Intervention by Arm

Among participants who initiated acupuncture, the mean number of treatments was 8.0 for group and 8.1 for individual (*p* = 0.56). The majority (63.7%) attended 8 or more treatments; there were no differences in failure to initiate acupuncture (7.7% individual vs. 11% group; *p* = 0.12) or number of sessions attended by study arm (see Table 2).

**Table 2:** Treatment Participation by Study Arm

	Group (*n* = 346)	Individual (*n* = 360)	Total (*n* = 779)	*p* value
Mean (SD)	8.0 (3.4)	8.1 (3.4)	8.1 (3.4)	0.56
Median (Q1, Q3)	9 (6, 11)	9 (6, 11)	9 (6, 11)	
No. of treatments				
0	43 (11%)	30 (7.7%)	73 (9.4%)	
1–7	126 (36.4%)	130 (36.1%)	256 (36.3%)	0.93
≥ 8	220 (63.6%)	230 (63.9%)	450 (63.7%)	

### Primary Outcome

Table 3 summarizes results for the primary outcome for both the ITT and PP samples. The PP sample included participants (63.7% of those who initiated treatment, *N* = 450) who attended a “full course” of treatment, defined as 8 or more treatment sessions as based on expert opinion and large meta-analyses.^33, 72^

**Table 3:** Primary Outcome: BPI Pain Interference

Outcome measure	Intent to treat			Per protocol (> 8 treatments)		
	Group	Individual	Between group difference	Group	Individual	Between group difference
Baseline						
*N*	385	385	N/A	219	229	N/A
Mean (SD)	6.0 (2.7)	6.1 (2.7)	0.15 (*p* = 0.44)	5.8 (2.6)	5.8 (2.8)	0.02 (*p* = 0.94)
Range (min–max)	0–10	0–10	N/A	0–10	0–10	N/A
12 weeks						
*N*	279	297	N/A	194	209	N/A
Mean (SD)	5.1 (3.0)	4.8 (3.1)	− 0.33 (*p* = 0.19)	4.6 (2.9)	4.5 (3.1)	− 0.08 (*p* = 0.79)
Mean change at 12 weeks from baseline (SD)	− 0.8 (2.6)	− 1.2 (2.6)	− 0.37 95% CI (− 0.77, 0.30)	− 1.1 (2.6)	−1.3 (2.7)	− 0.16 95% CI (− 0.65, 0.32)
Responders (> 30% improvement)	82 (30.3%)	108 (37.5%)	7.2% 95% CI (− 0.6%, 15.1%)	65 (34.4%)	81 (39.7%)	5.3% 95% CI (− 4.2%, 14.9%)
24 weeks						
*N*	276	292	N/A	186	202	N/A
Mean (SD)	5.3 (2.9)	5.1 (3.1)	− 0.23 (*p* = 0.73)	5.1 (3.0)	4.7 (3.1)	− 0.36 (*p* = 0.44)
Mean change at 24 weeks from Baseline (SD)	− 0.7 (2.5)	− 1.0 (2.5)	− 0.25 95% CI (− 0.66, 0.17)	− 0.8 (2.5)	− 1.1 (2.5)	− 0.31 95% CI (− 0.79, 0.17)
Responders (> 30% improvement)	77 (28.7%)	99 (35.0%)	6.3% 95% CI (− 1.5%, 14.0%)	56 (30.8%)	78 (39.6%)	8.8% 95% CI (− 0.8%, 18.4%)

Per ITT analysis, 37.5% of individual arm and 30.3% of group arm participants had > 30% improvement in pain interference at 12 weeks (*d* = 7.2%, 95% CI − 0.6%, 15.1%). In the PP sample, the proportion was 39.7% of individual and 34.4% of group (*d* = 5.3%; 95% CI − 4.2%, 14.9%). Non-inferiority of group acupuncture for the primary outcome was not demonstrated in either the ITT or PP analyses since in both analyses the upper limits of the confidence intervals for the difference in response rates exceeded the non-inferiority margin of 10%. We also measured pain interference at 24 weeks to assess persistence of effect. In the ITT sample at 24 weeks, 35.0% of individual arm participants and 28.7% in the group arm had > 30% improvement in pain interference (*d* = 6.3%, 95% CI − 1.5%, 14.0%).

When multiple imputation was applied to address missing data, non-inferiority of group therapy could not be declared in either the ITT or PP analyses (supplementary Table c).

### Secondary Outcomes

#### Pain Severity

In the ITT analysis, 34.8% of individual and 30.5% of group participants had > 30% reduction in pain severity at 12 weeks (*d* = 4.3%, 95% CI − 3.3%, 11.9%). In the PP sample, the proportion was 39.2% of individual compared with 36.3% of group participants (*d* = 2.8%; 95% CI − 6.5%, 12.2%). Non-inferiority of group was not demonstrated in either analysis. About a quarter of both arms had > 30% reduction at 24 weeks (Table 4).

**Table 4:** Secondary Outcomes

BPI pain severity	Intent to treat			Per protocol (> 8 treatments)		
	Group	Individual	Between group difference	Group	Individual	Between group difference
Baseline						
*N*	389	386	N/A	220	228	N/A
Mean (SD)	6.8 (1.8)	6.8 (1.9)	0.01 (*p* = 0.96)	6.7 (1.8)	6.8 (1.9)	0.01 (*p* = 0.94)
Range	0–10	1.5–10	N/A	1.5–10	1.5–10	N/A
12 weeks						
*N*	285	301	N/A	201	213	N/A
Mean (SD)	5.7 (2.5)	5.4 (2.7)	− 0.29 (*p* = 0.17)	5.3 (2.4)	5.1 (2.7)	− 0.19 (*p* = 0.45)
Mean change at 12 weeks from Baseline (SD)	− 1.1 (2.1)	− 1.4 (2.8)	− 0.26 95% CI (− 0.61, 0.09)	− 1.4 (2.2)	− 1.7 (2.2)	− 0.23 95% CI (− 0.65, 0.18)
Responders (> 30% improvement)	87 (30.5%)	104 (34.8%)	4.3% 95% CI (− 3.3%, 11.9%)	73 (36.3%)	83 (39.2%)	2.8% 95% CI (− 6.5%, 12.2%)
24 weeks						
*N*	286	294	N/A	196	204	N/A
Mean (SD)	6.0 (2.6)	5.8 (2.6)	− 0.18 (*p* = 0.72)	5.7 (2.5)	5.5 (2.5)	− 0.17 (*p* = 0.73)
Mean change at 24 weeks from baseline (SD)	− 0.8 (2.3)	− 1.1 (2.2)	− 0.23 95% CI (− 0.59, 0.13)	− 1.0 (2.3)	− 1.2 (2.3)	− 0.17 95% CI (− 0.62, 0.25)
Responders (> 30% improvement)	65 (22.8%)	74 (25.4%)*	2.6% 95% CI (− 4.4%, 9.6%)	48 (24.5%)	56 (27.7%)	3.2% 95% CI (− 5.4%, 11.9%)
**PROMIS: Physical health**						
Baseline						
*N*	385	387	N/A	217	228	N/A
Mean (SD)	34.8 (7.2)	34.8 (7.7)	0.05 (*p* = 0.92)	35.3 (7.1)	35.2 (7.6)	− 0.04 (*p* = 0.96)
12 Weeks						
*N*	295	308	N/A	207	219	N/A
Mean (SD)	38.5 (8.4)	38.7 (8.3)	0.21 (*p* = 0.76)	39.4 (8.3)	39.6 (8.1)	0.22 (*p* = 0.78)
Mean change at 12 weeks from baseline (SD)	3.6 (7.3)	3.6 (6.5)	0.08 95% CI (− 0.97, 1.13)	3.8 (7.1)	4.2 (6.4)	0.36 95% CI (− 0.87, 1.59)
Responders (≥ 2-point improvement)	173 (59.5%)	193 (63.1%)	3.6% 95% CI (− 4.2%, 11.4%)	125 (61.3%)	147 (67.7%)	6.5% 95% CI (− 2.7%, 15.6%)
24 weeks						
*N*	287	297	N/A	196	206	N/A
Mean (SD)	37.2 (8.7)	37.8 (8.5)	0.60 (*p* = 0.4)	38.2 (8.7)	38.7 (8.1)	0.54 (*p* = 0.52)
Mean change at 24 weeks from baseline (SD)	2.4 (7.3)	2.7 (6.4)	0.38 95% CI (− 0.69, 1.45)	2.5 (7.1)	3.1 (6.1)	0.64 95% CI (− 0.61, 1.90)
Responders (≥ 2-point improvement)	142 (50.2%)	164 (55.4%)	5.2% 95% CI (− 2.9%, 13.4%)	96 (49.7%)	121 (59.0%)	9.3% 95% CI (− 0.5%, 19.0%)
**PROMIS: Mental health**						
Baseline						
*N*	385	384	N/A	219	226	N/A
Mean (SD)	42.8 (9.8)	42.4 (9.6)	− 0.40 (*p* = 0.57)	43.6 (9.6)	43.5 (9.7)	− 0.09 (*p* = 0.92)
12 weeks						
*N*	291	308	N/A	204	218	N/A
Mean (SD)	43.8 (9.4)	44.5 (9.6)	0.76 (*p* = 0.33)	44.4 (9.2)	45.2 (9.3)	0.78 (*p* = 0.39)
Mean change at 12 weeks from baseline (SD)	1.1 (8.4)	1.5 (7.1)	0.48 95% CI (− 0.65, 1.61)	0.8 (8.0)	1.4 (6.6)	0.66 95% CI (− 0.61, 1.93)
Responders (≥ 5-point improvement)	75 (26.0%)	84 (27.6%)	1.6% 95% CI (− 5.6%, 8.7%)	48 (23.6%)	57 (26.5%)	2.9% 95% CI (− 5.4%, 11.2%)
24 weeks						
*N*	288	297	N/A	198	206	N/A
Mean (SD)	43.3 (9.9)	44.1 (9.4)	0.78 (*p* = 0.33)	44.1 (9.7)	45 (9.0)	0.88 (*p* = 0.35)
Mean change at 24 weeks from baseline (SD)	0.8 (7.5)	1.3 (6.9)	0.52 95% CI (− 0.56, 1.61)	0.4 (7.2)	1.4 (6.6)	0.94 95% CI (− 0.30, 2.18)
Responders (≥ 5-point improvement)	72 (25.3%)	86 (29.2%)	3.9% 95% CI (− 3.4%, 11.1%)	45 (22.8%)	59 (28.9%)	6.1% 95% CI (− 2.5%, 14.6%)

#### PROMIS 10 Global Health^64^

In the ITT sample, baseline physical health T-score was 34.8 in both arms. In the ITT analysis, 63.1% of individual arm and 59.5% of group participants had clinically important improvement (defined as a two-point change in mean T-score)^73, 74^ at 12 weeks for physical health (*d* = 3.6%, 95% CI − 4.2%, 11.4%). At 24 weeks 55.4% of individual and 50.2% of group still reported clinically important improvement (*d* = 5.2%, 95% CI − 2.9%, 13.4%). In the PP sample, 67.7% of individual and 61.3% of group had 2 point or greater improvement at 12 weeks (*d* = 6.5%, 95% CI − 2.7%, 15.6%); at 24 weeks, 59.0% of individual arm versus 49.7% of group participants still reported response (*d* = 9.3%, 95% CI − 0.5%, 19.0%).

Minimal changes were observed in mean mental health scores in both arms, in both the ITT and PP samples. As no minimal important difference for the mental health subscale has been established, we used a change of one half standard deviation (5 points) to define “response.” About a quarter of both samples had clinically important improvement at 12 weeks.

#### Opiate Use

Based on EMR data, of the 706 participants, 191 had at least one opioid analgesic prescription during the study period. The proportion of patients with an opioid prescription in the ITT sample was significantly higher 12 weeks prior to randomization than in the period 4–16 weeks after in the individual arm (16.4% vs. 11.0%, *p* = 0.003), but not in the group arm (13.1% vs. 14.3%, *p* = 0.39). In the PP sample, individual arm patients had a decrease in the average daily dose of opioid (in morphine milliequivalents) before and after randomization (39.1 mg vs. 30.4 mg, *p* = 0.05) but those in group treatment did not (13.5 mg vs. 15.2 mg, *p* = 0.27). Results are similar when comparing the longer time frames of 24 weeks prior to 4–28 weeks post-randomization (see supplementary table d). By self-report, for the total sample, there was no difference in the proportion using an opiate pain reliever in the past 7 days at baseline versus 12 weeks in either sample (supplemental Table e). There is a modest decrease in the PP sample only in mean days of use in the past week (supplemental Table f).

### Adverse Events

No serious adverse events (AE) were reported in either individual or group acupuncture cohorts. Fifteen non-serious AEs were documented including transient pain at a needle site, short-term exacerbation of chronic pain condition, dizziness or nausea, with one participant fainting.^49^

## DISCUSSION

We found clinically significant improvement in pain interference in both group and individual arms for a substantial proportion of participants at 12 weeks in both our ITT and PP analyses. Pain severity also showed clinically meaningful improvement in over 30% of participants in both arms, and global physical health in roughly 60%. Non-inferiority of group to individual acupuncture was not demonstrated for either pain interference or severity at 12 weeks; individual treatment was consistently slightly better than group. Regarding opiate use, based on EMR data, opiate prescriptions declined in the individual arm but not in the group arm when comparing the 12 weeks pre-intervention to the period 4–16 weeks post-intervention.

Although our response rates in both arms were slightly lower than the 40–50% response seen in a large individual patient data meta-analyses,^33, 72^ and although we did not demonstrate non-inferiority of group treatment, our results suggest that both individual and group acupuncture can be offered safely in the community health center setting, and that a substantial proportion of patients with chronic pain will have clinically significant improvement. In light of the many recent guidelines documents supporting the use of acupuncture as part of comprehensive pain care and to mitigate opioid risks,^34–36, 75, 76^ this is an important finding. We also found that acupuncturists saw on average 1.9 patients per hour in group sessions compared with 1.4 per hour in individual (35% increase) suggesting a possible cost advantage to the group model. This may be an underestimate of the increased efficiency of the group model: in well-managed practice settings (rather than a clinical trial) acupuncturists would typically see 2 patients per hour for individual treatment and 4 patients per hour for group treatment. Finally, group care provided in a common, multipurpose room reduces the cost of utilization of individual medical exam rooms, which are typically in high demand in these settings.

Regarding acceptability of group treatment, there was no difference in the number of sessions attended for participants in the two arms, or any difference in treatment initiation after randomization. Participants who might have had an initial preference for individual treatment reliably initiated and continued treatment in the group setting. In qualitative interviews with participants in both study arms, ^77^ we identified both positive (social interaction) and negative (privacy concerns, mixed-gender groups) elements, but none of these ultimately affected initiation and continuation of treatment.

A number of factors specific to our treatment setting and population make the positive response rates particularly meaningful. For both individual and group arms, delivery in busy community health centers presented challenges. The physical plant was designed for needs of primary care. Individual treatment occurred in medical exam rooms with tables not designed for a comfortable supine or prone position. Group sessions were scheduled in multi-purpose conference rooms with a table and chairs. Our population was also different in many ways from those in most clinical trials to date: participants often had multiple significant comorbidities, including depression, higher levels of disability and lower functional status, and significant socioeconomic and biopsychosocial challenges. These challenges may have contributed to the lower response rate. A recent trial of yoga versus physical therapy (PT) for chronic low back pain in an undeserved population found response rates very similar to those in our study (35%).^78^ Our previous study of individual acupuncture in this setting similarly found that roughly 1/3 of participants had a 30% or greater improvement in pain,^79^ suggesting that this may be a more typical response in this population. Regarding the difference in outcomes between group and individual arms, it is possible that constraints on the physical environment may have contributed, in particular, the challenge of treating patients seated in chairs with limited capacity for accessing acupuncture points on the trunk and upper legs. A recent pilot trial which provided group acupuncture in a more optimal setting found a larger proportion of patients experiencing a clinically significant reduction in pain and depression.^46^

### Study Limitations

The most significant limitation of this study was that due to resource limitations, our pragmatic trial design did not include a third arm representing usual care alone; thus, we cannot definitively attribute the participants’ benefit to the acupuncture treatment. However, the benefit of acupuncture compared with both placebo and usual care has been shown elsewhere in large individual patient data meta-analyses.^33, 72^ A second limitation was the suboptimal physical setting of group acupuncture delivery. This could have biased results away from non-inferiority of group over individual. In future implementation, this limitation could be mitigated by the addition of more comfortable chairs and one or two mobile treatment tables. The ITT group includes 73 people who were randomized but never initiated treatment, biasing ITT results toward less effective overall. There were several limitations to use of EMR data to examine opioid analgesic use. First, participants possibly obtained prescriptions outside of our health system which would have been unavailable for analysis. Second, the assumption was made that participants used all as-needed doses available to them by prescription, which may not be accurate. Although these limitations would theoretically apply equally to both groups, based on the fact that the standard deviation was extremely wide and that opioid utilization was a secondary outcome for which we were not adequately powered, our findings on opioids should be seen as hypothesis-generating for future research rather than definitive.

## CONCLUSIONS

Our results demonstrate that individual and group acupuncture can be offered safely in the community health center setting, that acceptability to patients and clinicians is very high, and that a substantial proportion of patients with chronic pain will have clinically significant improvement in both pain and overall physical health. Based on these results, acupuncture therapy should be offered as part of pain care to underserved populations in the primary care setting. Non-inferiority of group treatment was not demonstrated, suggesting that further research is needed on the optimal strategy for delivering group acupuncture in this context to consider it as effective as individual treatment.

## Electronic Supplementary Material

ESM 1 (DOCX 35 kb)
