# Supplementary material for: Individual vs. Group Delivery of Acupuncture Therapy for Chronic Musculoskeletal Pain in Urban Primary Care—a Randomized Trial
Source: J Gen Intern Med. 2020 Feb 19;35(4):1227–37. doi: 10.1007/s11606-019-05583-6 (PMC7174252; doi:10.1007/s11606-019-05583-6)
Supplement: Supplementary file 1 — (DOCX 35 kb) [file 11606_2019_5583_MOESM1_ESM.docx]

**Supplemental Table a. Missing Pain Outcome Data for Each Assessment***

|  | **Pain Interference** | | **Pain Severity** | |
| --- | --- | --- | --- | --- |
|  | **Group (n=389)** | **Individual (n=390)** | **Group (n=389)** | **Individual (n=390)** |
| **Baseline** | 4 (1%) | 5 (1%) | 0 (0%) | 4 (1%) |
| **6 weeks** | 103 (26%) | 85 (22%) | 104 (27%) | 82 (21%) |
| **12 weeks** | 110 (28%) | 93 (24%) | 104 (27%) | 89 (23%) |
| **24 weeks** | 113 (29%) | 98 (25%) | 103 (26%) | 96 (25%) |
| *due to no survey submitted or incomplete data for the pain outcome | | | | |

### Supplemental Table b. Comparison of baseline demographics for those with missing data for primary outcome at 12 weeks.

| **Demographic Variable** | | **With missing data** | **Without missing data** | **Total** | **P-value** |
| --- | --- | --- | --- | --- | --- |
|  |  | **(N=209)** | **(N=570)** | **(N=779)** |  |
| **Age,** Mean (SD) | | 52.81 (13.75) | 55.64 (13.13) | 54.88 (13.35) | 0.01 |
| **Sex, n (%)** | |  |  |  | 0.87 |
|  | Female | 169 (80.9%) | 458 (80.4%) | 627 (80.5%) |  |
|  | Male | 40 (19.1%) | 112 (19.7%) | 152 (19.5%) |  |
| **Spoken Language, n (%)** | | | | | 0.27 |
|  | English | 152 (79.2%) | 430 (78.0%) | 582 (78.3%) |  |
|  | Spanish | 39 (20.3%) | 121 (22.0%) | 160 (21.5%) |  |
|  | Other | 1 ( 0.5%) | 0 (0.0%) | 1 ( 0.1%) |  |
| **Ethnicity**, n (%) | |  |  |  | 0.13 |
|  | Hispanic/Latino(a) | 126 (60.6%) | 317 (55.6%) | 443 (56.9%) |  |
|  | Non-Hispanic | 82 (39.4%) | 245 (43.0%) | 327 (42.0%) |  |
|  | Don’t Know | 0 (0.0%) | 8 ( 1.4%) | 8 ( 1.0%) |  |
| **Race**, n (%) | |  |  |  | 0.10 |
|  | American Indian/Native | 15 ( 7.5%) | 24 ( 4.4%) | 39 ( 5.2%) |  |
|  | Asian | 5 ( 2.5%) | 5 ( 0.9%) | 10 ( 1.3%) |  |
|  | Black/African American | 75 (37.3%) | 200 (36.2%) | 275 (36.5%) |  |
|  | White | 26 (12.9%) | 78 (14.1%) | 104 (13.8%) |  |
|  | Multiracial | 30 (14.9%) | 66 (11.9%) | 96 (12.7%) |  |
|  | Other | 50 (24.9%) | 180 (32.5%) | 230 (30.5%) |  |
| **Working status**, n (%) | | | | | 0.67 |
|  | Unable to work due to disability | 75 (35.9%) | 215 (37.7%) | 290 (37.2%) |  |
|  | Unemployed | 31 (14.8%) | 71 (12.5%) | 102 (13.1%) |  |
|  | Other (Employed, retired, etc.) | 103 (49.3%) | 284 (49.8%) | 387 (49.7%) |  |
| **Highest Schooling Completed**, n (%) | | | | | <0.0001 |
|  | HS education or less | 142 (67.9%) | 283 (49.6%) | 425 (54.6%) |  |
|  | Some College or more | 67 (32.1%) | 287 (50.4%) | 354 (45.4%) |  |
| **Household Income Support,** n (%) | | | | | 0.28 |
|  | Any Support | 64 (31.2%) | 153 (27.3%) | 217 (28.3%) |  |
|  | No Support | 141 (68.8%) | 408 (72.7%) | 549 (71.7%) |  |

### Supplemental Table c: ITT and PP analyses of primary outcome using multiple imputation to handle missing data

|  | **Outcome Measure** | **Intent to Treat (n=779)** | | **Per Protocol (>8 treatments) (n=450)** | |
| --- | --- | --- | --- | --- | --- |
|  |  | **Between Group Difference*** | **95% CI** | **Between Group Difference*** | **95% CI** |
| **12 Weeks** | | | | | |
|  | **Pain severity**  **(≥ 30% improvement)** | 0.05 | [-0.03, 0.12] | 0.03 | [-0.06, 0.13] |
|  | **Pain interference**  **(≥ 30% improvement)** | 0.06 | [-0.02, 0.14] | 0.04 | [-0.06, 0.13] |
|  | **PROMIS physical health (2 points change)** | 0.03 | [-0.05, 0.10] | 0.06 | [-0.03, 0.15] |
|  | **PROMIS mental health**  **(5 points change)** | 0.03 | [-0.04, 0.09] | 0.03 | [-0.05, 0.11] |
| **24 Weeks** | | | | | |
|  | **Pain severity (≥ 30% improvement)** | 0.03 | [-0.04, 0.09] | 0.04 | [-0.04, 0.12] |
|  | **Pain interference (≥ 30% improvement)** | 0.04 | [-0.03, 0.11] | 0.07 | [-0.03, 0.16] |
|  | **PROMIS physical health (2 points change)** | 0.04 | [-0.04, 0.12] | 0.08 | [-0.02, 0.17] |
|  | **PROMIS mental health (5 points change)** | 0.04 | [-0.03, 0.11] | 0.06 | [-0.03, 0.14] |
|  |  |  |  |  |  |
|  | ***(Individual - Group)** |  |  |  |  |

Variables included in the multiple imputation model for the pain severity and interference outcomes were age, gender, place of birth, race/ethnicity, household income, education, insurance, marital status, work status, and source of pain, as well as the auxiliary variables, baseline and 12-week scores for secondary outcomes including PROMIS physical and mental health and PHQ9 score. Sensitivity analyses were also performed by varying the covariates included in the imputation model.

**Supplemental Table d. Proportion with Opioid Prescriptions (EMR-data), Intent to Treat and Per Protocol**

| **Proportion with Opioid Prescriptions** | | **Intent to Treat** | |  | **Per Protocol (>8 treatments)** | | |
| --- | --- | --- | --- | --- | --- | --- | --- |
|  |  | **Group** | **Individual** | **Total** | **Group** | **Individual** | **Total** |
| **Prescription 12 weeks prior to randomization** | |  |  |  |  |  |  |
|  | N | 51/389 | 64/390 | 115/779 | 26/220 | 35/230 | 61/450 |
|  | % | 13.1% | 16.4% | 14.8% | 11.8% | 15.2% | 13.6% |
| **Prescription 4-16 weeks after randomization** | |  |  |  |  |  |  |
|  | N | 57/389 | 43/390 | 100/779 | 28/220 | 22/230 | 50/450 |
|  | % | 14.7% | 11.0% | 12.8% | 12.7% | 9.6% | 11.1% |
|  | Difference between time periods | +1.6% | -5.4% |  | +0.90% | -5.6% |  |
|  | P-value | 0.39 | 0.003 | 0.13 | 0.68 | 0.12 | .014 |
| **Prescription 24 weeks prior to randomization** | |  |  |  |  |  |  |
|  | N | 66/389 | 73/390 | 139/779 | 31/220 | 41/230 | 72/450 |
|  | % | 17.0% | 18.7% | 17.8% | 14.1% | 17.8% | 16.0% |
| **Prescription 4-28 weeks after randomization** | |  |  |  |  |  |  |
|  | N | 65/389 | 55/390 | 120/779 | 32/220 | 28/230 | 60/450 |
|  | % | 16.7% | 14.1% | 15.4% | 14.5% | 12.2% | 13.3% |
|  | Difference between time periods | -0.3% | -4.6% | -2.4% | +0.4% | -5.6% | -2.7% |
|  | P-value | 0.896 | 0.022 | 0.084 | 0.853 | 0.033 | 0.14 |

**Supplemental Table e. Self-Report Opiate Use in the Last 7 days, all participants**

|  |  | **Baseline** | **12 Weeks** | **P-value** |
| --- | --- | --- | --- | --- |
| **Intent to Treat** | | | | |
|  | N | 765 | 596 |  |
|  | Self-report Opiate use in Past 7 days (yes, %) | 163 (21.3%) | 108 (18.1%) | 0.17 * |
|  | Mean days used in the past week (SD) | 4.7 (3.2) | 4.6 (3.2) | 0.03 ^ |
| **Per Protocol** | | | | |
|  | N | 441 | 422 |  |
|  | Self-report Opiate use in Past 7 days (yes, %) | 81 (18.4%) | 65 (15.4%) | 0.12 * |
|  | Mean days used in the past week (SD) | 4.3 (3) | 3.9 (3) | 0.0026 ^ |

* McNemar Test

^ Wilcoxon signed ranked test

**Supplemental Table f. Mean Morphine Equivalents (MME) from EMR-data, Intent to Treat and Per Protocol**

| **MME** | | **Intent to Treat** | |  | **Per Protocol (>8 treatments)** | | |
| --- | --- | --- | --- | --- | --- | --- | --- |
|  |  | **Group** | **Individual** | **Total** | **Group** | **Individual** | **Total** |
| **12 weeks prior to randomization** | |  |  |  |  |  |  |
|  | N | 389 | 390 | 779 | 220 | 230 | 450 |
|  | Mean (SD) | 16.5 mg (29.1) | 32.4 mg (85.5) | 24.4 mg (58.4) | 13.5 mg (26.3) | 39.1 mg (109.1) | 27.2 mg (82.5) |
| **4-16 weeks after randomization** | |  |  |  |  |  |  |
|  | N | 389 | 390 | 779 | 220 | 230 | 450 |
|  | Mean (SD) | 15.1 mg (27.4) | 30.7 mg (77.6) | 22.9 mg (58.4) | 15.2 mg (30.2) | 30.5 (90.6) | 23.4 mg (69.5) |
|  | Difference between time periods | -1.40 mg (19.12) | -1.73 mg (42.42) | -1.56 (32.8) | +1.71 (13.34) | -8.68 (37.24) | -3.9 (29.1) |
|  | P-value | 0.54 | 0.20 | 0.62 | 0.27 | 0.05 | 0.43 |
| **24 weeks prior to randomization** | |  |  |  |  |  |  |
|  | N | 389 | 390 | 779 | 220 | 230 | 450 |
|  | Mean (SD) | 15.24 mg (26.7) | 28.6 mg (74.3) | 21.9 mg (56.0) | 12.2 mg (25.5) | 32.8 mg (94.1) | 23.2 (71.5) |
| **4-28 weeks after randomization** | |  |  |  |  |  |  |
|  | N | 389 | 390 | 779 | 220 | 230 | 450 |
|  | Mean (SD) | 14.51 mg (25.5) | 27.8 mg (69.0) | 21.1 mg (52.2) | 14.1 mg (28.3) | 28.2 mg (82.6) | 21.6 mg (63.5) |
|  | Difference between time periods | -0.74 mg (15.4) | -0.77 mg (29.1) | -0.75 mg (23.2) | 1.97mg  (9.98) | -4.7mg  (23.6) | -1.6% (18.7) |
|  | P-value | 0.27 | 0.38 | 0.95 | 0.09 | 0.15 | 0.92 |
